# Emergent superconductivity in an iron-based honeycomb lattice initiated by pressure-driven spin-crossover

**DOI:** 10.1038/s41467-018-04326-1

**Published:** 2018-05-15

**Authors:** Yonggang Wang, Jianjun Ying, Zhengyang Zhou, Junliang Sun, Ting Wen, Yannan Zhou, Nana Li, Qian Zhang, Fei Han, Yuming Xiao, Paul Chow, Wenge Yang, Viktor V. Struzhkin, Yusheng Zhao, Ho-kwang Mao

**Affiliations:** 1 Center for High Pressure Science and Technology Advanced Research (HPSTAR), 100094 Beijing, China; 2 HPSynC, Geophysical Laboratory, Carnegie Institution of Washington, Argonne, IL 60439 USA; 3 Geophysical Laboratory, Carnegie Institution of Washington, Washington, DC 20015 USA; 4 HPCAT, Geophysical Laboratory, Carnegie Institution of Washington, Argonne, IL 60439 USA; 5 College of Chemistry and Molecular Engineering, Peking University, 100871 Beijing, China; 6 College of Chemistry and Chemical Engineering, Chongqing University, 400044 Chongqing, China; 7 Institute of Nanostructured Functional Materials, Huanghe Science and Technology College, 450006 Zhengzhou, China; 8 Southern University of Science and Technology, 518055 Shenzhen, China

## Abstract

The discovery of iron-based superconductors (FeSCs), with the highest transition temperature (*T*_c_) up to 55 K, has attracted worldwide research efforts over the past ten years. So far, all these FeSCs structurally adopt FeSe-type layers with a square iron lattice and superconductivity can be generated by either chemical doping or external pressure. Herein, we report the observation of superconductivity in an iron-based honeycomb lattice via pressure-driven spin-crossover. Under compression, the layered FeP*X*_3_ (*X* = S, Se) simultaneously undergo large in-plane lattice collapses, abrupt spin-crossovers, and insulator-metal transitions. Superconductivity emerges in FePSe_3_ along with the structural transition and vanishing of magnetic moment with a starting *T*_c_ ~ 2.5 K at 9.0 GPa and the maximum *T*_c_ ~ 5.5 K around 30 GPa. The discovery of superconductivity in iron-based honeycomb lattice provides a demonstration for the pursuit of transition-metal-based superconductors via pressure-driven spin-crossover.

## Abstract (web summary)

Up to now, all iron-based high-*T*_c_ superconductors contain a square iron lattice. Here, Wang et al. report the observation of superconductivity in an iron honeycomb lattice accompanied with pressure-driven spin-crossover, in-plane lattice collapse and insulator-metal transition.

## Introduction

Since the discovery of 26 K superconductivity (SC) in LaO_1*−x*_F_*x*_FeAs in 2008^1^, the Fe-based superconductors (FeSCs) have attracted enormous research interest, owing to their rich compositional and structural varieties^1–12^. The rapid development on this new SC family has led to the highest critical temperature (*T*_c_) up to 55 K in F-doped SmFeAsO^2^ and the discovery of a large number of FeSCs with various structure types^3,5–12^. Similar to the high-*T*_c_ cuprate family unexceptionally adopting square CuO_2_ layers, all of these FeSCs structurally possess infinite antifluorite-like Fe_2_*X*_2_ layers comprising the stacks of edge-sharing Fe*X*_4_ tetrahedra (*X* denotes a pnictide or a chalcogenide element). Therefore, it is widely believed that the common Fe_2_*X*_2_ layer is the essential building unit for the rational structure design of FeSCs, similar in nature to the CuO_2_ unit in cuprate SCs. And this viewpoint is verified by the discovery of high-*T*_c_ SC in a large number of FeSe-derived layered compounds such as *A*_*x*_Fe_2−*y*_Se_2_ (*A* = alkali metals)^9,10^, Li_*x*_(NH_2_)_*y*_(NH_3_)_1−*y*_Fe_2_Se_2_^11^, and (Li_0.8_Fe_0.2_)OHFeSe^12^. Another key feature of FeSCs is a well-recognized fact that in most cases the high-*T*_c_ SC can emerge by suppressing the long-range antiferromagnetic (AFM) order in the stoichiometric parent compounds. Experimentally, two general routes are adopted to achieve this purpose: first, chemical doping or substitution, such as F doping in REFeAsO^1^ (RE = rare earth elements) and interlayer cation doping (alkali metals or NH_3_)^9–11^; second, application of chemical or external pressure, such as the replacement of *X* in Fe_2_*X*_2_ by congeners with bigger or smaller atomic radii or the substitution of smaller RE ions for La ions^2^.

Under applied high pressure (HP), materials undergo direct structural evolutions including the shortening of metal-ligand bond length, the distortions in the nearest neighbor environment, and the introduction of stress. These structural evolutions make HP significant in the researches on SCs, and many unanticipated SCs have been discovered with this powerful tool. A recent famous example is the observation of conventional SC with high-*T*_c_ in hydrogen-rich systems, H_*x*_S, and PH_3_^13,14^, under HPs up to megabar scale. Besides, unexpected SC phase diagrams (e.g., MnP^15^, Tl_0.6_Rb_0.4_Fe_1.67_Se_2_, and K_0.8_Fe_1.7+*δ*_Se_2_^16^) and greatly enhanced *T*_c_ values in both cuprates and FeSCs (e.g., LaO_1−*x*_F_*x*_FeAs^17^, HgBa_2_Ca_2_Cu_3_O_8+*δ*_^18^, and *β*-Fe_1.01_Se^19^) have also been successfully achieved by pressure tuning. Moreover, applying external pressure to transition metal (TM) systems may also lead to other significant phenomena such as large-volume collapse^20–22^, spin-crossover (SCO)^22–24^, charge disproportionation^25^, and insulator-metal transition (IMT). Regarding the pressure-driven SCO, in which magnetic ions undergo high-to-low spin-state transition, it is possible to produce a nonmagnetic phase through the pressure-induced spin-quenching (*S* = 0). Keeping the relationship between magnetism and SC in mind, an adventurous but meaningful idea is: can we make new nonmagnetic FeSCs without the FeSe-type structure via pressure-driven spin-quenching? If so, Fe^2+^ with *d*^6^ electrical configuration in a sixfold-coordinated environment should be an ideal candidate to ensure a nonmagnetic HP phase with zero spin ground state (*S* = 2 to *S* = 0).

In the pursuit of abrupt pressure-driven SCOs in TM chalcogenides, we became aware of the essential role of the TM-sublattice dimensions in the cooperativeness of SCO^26,27^. We have discovered abrupt pressure-driven phase transitions accompanied by a high-to-low spin-state transition of Mn^2+^ in the transition from three-dimensional (3D) NaCl-type Mn*X* to two-dimensional (2D) MnP*X*_3_ (*X* = S, Se) with an Mn^2+^ honeycomb lattice. Subsequent experiments showed that similar cooperative SCO phenomenon also exists in the iron homologs FeP*X*_3_, in which Fe^2+^ ions (*d*^6^) arranged on a nearly perfect honeycomb lattice undergo a sharp magnetic-to-nonmagnetic transition (*S* = 2 to *S* = 0). This 2D system apparently meets all the above-mentioned prerequisites for the emergence of SC. In this letter, we report our discoveries in the course of HP studies of the layered compound FeP*X*_3_ (*X* = S, Se). Along with the pressure-driven SCO, in-plane lattice collapse, and semiconductor-to-metal transition, SC emerges around 3 ~ 5 K in the nonmagnetic HP phase of FePSe_3_. The physical property and the phase diagram of FeP*X*_3_ under HP are discussed along with the structural analyses results.

## Results

### Pressure-induced large-volume collapse

**Fig. 1:**
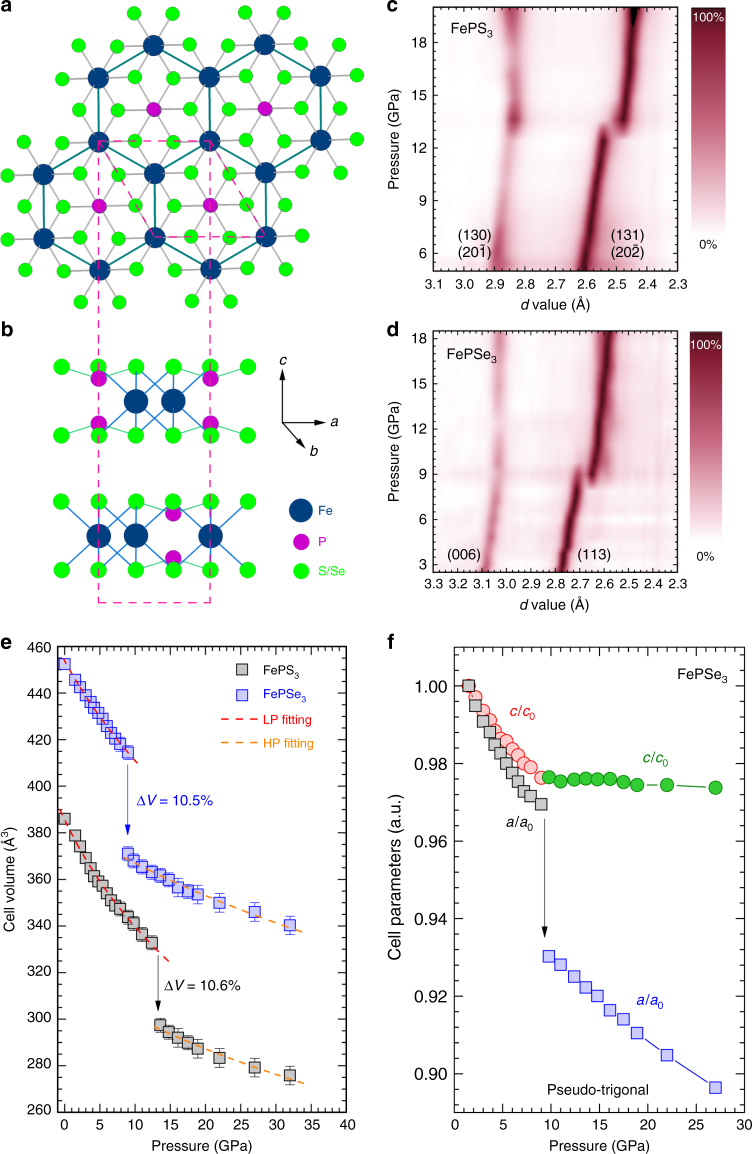
The crystal structure and pressure-induced phase transition of FeP*X*_3_. **a**, **b** Ambient crystal structure of FeP*X*_3_ (*X* = S, Se) viewed along *c*- and *a*/*b*-axis showing the layered structure feature and the Fe^2+^ honeycomb lattice. **c**, **d** Two-dimensional PXRD data showing abrupt pressure-induced changes of the (131) and (20-2) peaks for FePS_3_ and the (113) peak for FePSe_3_, respectively. **e** The derived cell volume values as a function of applied pressure for the LP and HP phases of FePS_3_ and FePSe_3_. The derived bulk moduli are: *B*_0_ = 61.1(2) and 162(9) GPa for LP and HP FePS_3_; *B*_0_ = 82.8(7) and 201(8) GPa for LP and HP FePSe_3_, respectively. Error bars represent ± S.D. **f** The cell parameters of FePSe_3_ with a pseudo-trigonal unit cell showing an in-plane collapse along with the pressure-induced phase transition

FeP*X*_3_ (*X* = S, Se) compounds adopt a 2D crystal structure with Fe^2+^ ions arranged in a nearly perfect honeycomb lattice and weak interlayer van der Walls interactions^28^. Figure 1a, b display the crystal structure of the low-pressure (LP) phase of FePS_3_ and FePSe_3_ with an ideal trigonal symmetry. Each layer is composed of two layers of S/Se atoms, octahedrally coordinated Fe^2+^ ions, and phosphorous pairs. All the Fe*X*_6_ octahedra share their edges with three neighbors to form the honeycomb lattice, and with P = P dimers locating in the center of the sixfold mesh. The shortest Fe–Fe distances in FeP*X*_3_ are ~3.44 Å for *X* = S and 3.62 Å for *X* = Se, respectively, and both compounds are narrow-band semiconductors at ambient conditions. Under compression, FeP*X*_3_ undergo isostructural or quasi-isostructural phase transitions at ~13 GPa for *X* = S and ~8 GPa for *X* = Se, respectively (Supplementary Fig. 1). The discontinuous shifts of the diffraction peaks ((113)/(20-2) of FePS_3_ and (113) of FePSe_3_, as shown in Fig. 1c, d) indicate abruptly shrinking of the lattice parameters concomitant with the pressure-driven phase transition, in particular, the collapses in the *ab*-plane lattice spacings.

Judging from the X-ray diffraction (XRD) patterns, monoclinic unit cells with the space group *C*2/*m* were adopted for the Le Bail fitting analyses for both FePS_3_ and FePSe_3_ (Supplementary Fig. 2). Figure 1e presents the evolution of the cell volumes of FeP*X*_3_ as a function of applied pressure. Large cell volume decreases (10.5% for *X* = S and 10.6% for *X* = Se) dominate the *P*–*V* profiles along with the first-order phase transitions, indicating remarkable changes of the atomic arrangement. Such large-volume collapses (>5%) are usually associated with the pressure-driven SCO on the origin^21,22^. The decrease of Fe^2+^ ionic radii from 0.78 Å (high spin (HS), *S* = 2) to 0.61 Å (low spin (LS), *S* = 0) results in the shortening of Fe-(S/Se) bond lengths and the possible symmetry lowering of the hexagonal lattice. To sententiously analysis the anisotropic compressibility of FeP*X*_3_, a pseudo-trigonal space group *R*-3 is adopted for the XRD refinements of FePSe_3_ (Fig. 1f). The dramatic decreases of *a* and *b* during the phase transition clearly indicate that the volume collapses origin from the shrinkages of the Fe^2+^ honeycomb lattices, which also indicate the formation of in-plane intermetallic Fe–Fe bonding. Similar in-plane lattice collapse is also observed in FePS_3_ with monoclinic symmetry (Supplementary Fig. 3).

### Pressure-induced spin-crossover

**Fig. 2:**
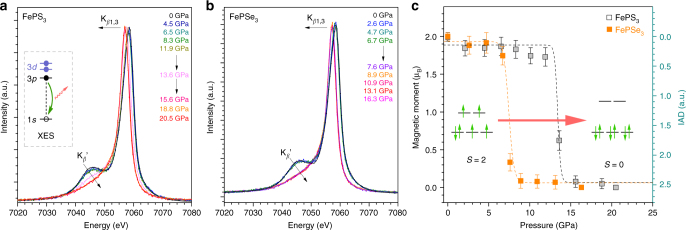
XES data of FePS_3_ and FePSe_3_ showing the spin-crossover of Fe^2+^ under compression. **a**, **b** Fe K_*β*_ XES of FePS_3_ and FePSe_3_ as a function of applied pressure. Inset of **a** shows the XES process from 3*p* to 1*s* orbital, and the relationship with 3*d* spin state. **c** The derived IAD values and corresponding magnetic moment numbers of FePS_3_ and FePSe_3_ as a function of pressure. Error bars represent ± S.D. estimated for the IAD analyses

We have proposed that the pressure-driven cooperative SCO, i.e., large-volume collapse accompanied with SCO and IMT, should be a universal behavior of 3*d* TM chalcogenides^26,27^. FeP*X*_3_, as an ideal low-dimensional confined system similar to MnP*X*_3_^27^, thus is highly expected to achieve a nonmagnetic HP phase via pressure-driven cooperative SCO. In situ X-ray emission spectroscopy (XES)^23^ measurements were performed to study the spin-state transitions of FeP*X*_3_ under HP and the results are presented in Fig. 2a, b. It is generally recognized that the K_*β*_ lines are the characteristic emissions originated from the 3*p* → 1*s* transition, and the shapes of K_*β*_ lines are determined by the interactions between the 3*p* core hole and the partially filled 3*d* shell electrons. Therefore, it allows qualitative distinction of the HS/LS states from the relative intensities of satellite K_*β*_′ and the peak positions of K_*β*1,3_. In the LP phases of FeP*X*_3_, well-defined K_*β*_′ satellite peaks are observed with a HS state (*S* = 2). Concomitant with the structural phase transition, the K_*β*_′ peaks drop suddenly and the K_*β*1,3_ lines shift to lower energy correspondingly, both of which indicate the occurrence of SCO of Fe^2+^ within the honeycomb lattice.

Quantitative analysis of the XES data has been conducted using the integrals of the absolute values of the difference spectra (IAD) method^29^, and the differential curves are provided in Supplementary Fig. 4. The pressure dependence of the Fe^2+^ spin state in FeP*X*_3_ is presented in Fig. 2c. At ambient conditions, the two compounds have a HS state of Fe^2+^ (3*d*^6^) with *S* = 2 (IAD = 0). The HS state of Fe^2+^ is stable until the applied pressure exceeds the LP-to-HP structural transition points. The IAD values of FeP*X*_3_ increase to ~2.5 (*S* = 0) abruptly, indicating a complete collapse of the Fe^2+^ spin moments. Like the pressure effect on MnP*X*_3_ system^27^, the abrupt pressure-driven SCO in FeP*X*_3_ is attributed to their 2D crystal structures, where the spins located on the honeycomb lattice can more collectively communicate with neighboring spins than those on 3D lattices.

### Pressure-induced semiconductor-to-metal transition

**Fig. 3:**
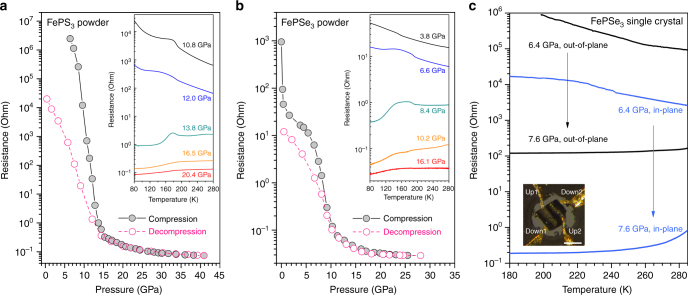
Pressure-induced semiconductor-to-metal transitions in FePS_3_ and FePSe_3_. **a**, **b** Electrical resistances of compressed powder of FeP*X*_3_ (*X* = S, Se) as a function of pressure. Insets of **a**, **b** show the temperature dependence (*R*–*T*) of the resistance of FePS_3_ and FePSe_3_ under high pressure, respectively. **c** Electrical resistance measurements on a FePSe_3_ single crystal showing an in-plane metallization behavior. Inset of **c** shows the photograph of the FePSe_3_ single crystal with four Au probes inside a DAC. The scale bar is 100 μm

In situ transport measurements of FeP*X*_3_ show IMT along with the pressure-driven structural phase transition and SCO (as shown in Fig. 3). During the IMT process, the electrical resistances of FeP*X*_3_ drop by more than five orders of magnitude at room temperature. The *R*–*T* curves under HP also indicate a change from a semi-conductive behavior of the LP phases to metallic behavior of the HP phases, concomitant with the structural and electronic configuration transitions. Since the Fe–Fe intermetallic bonding can only form in the honeycomb layer, it is reasonable to see that the HP phases exhibit a bad-metal and an anisotropic transporting behavior. The conductivity measurements on the single crystal of FePSe_3_ confirm that the in-plane metallization contributes most to the overall IMT phenomenon (Fig. 3c). What needs to be pointed out here is, all the data obtained in our transport measurements are original resistances rather than absolute resistivity, which may be converted to each other by normalizing the ambient resistivity values inside and outside the diamond anvil cell (DAC).

### Observation of superconductivity

**Fig. 4:**
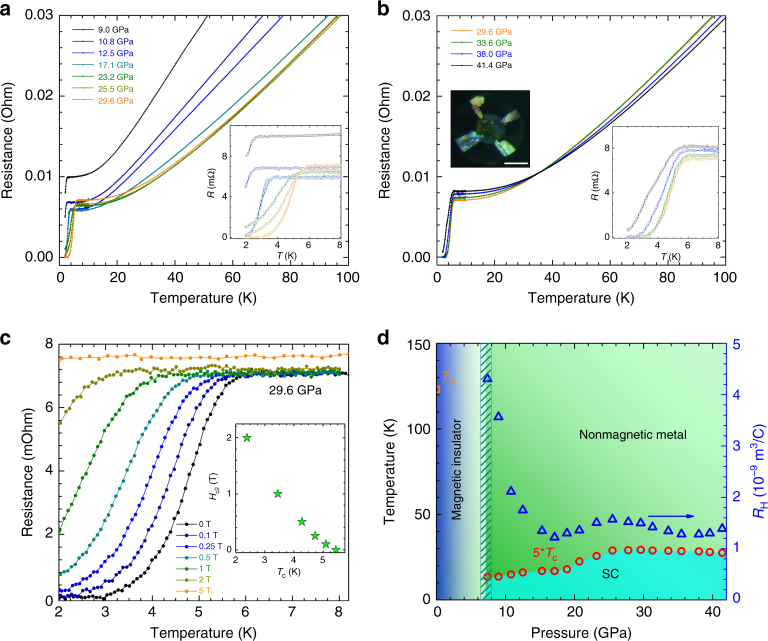
Emergence of superconductivity under high pressure and temperature–pressure phase diagram in FePSe_3_. **a** The in-plane electrical resistivity of FePSe_3_ single crystal as a function of temperature, and applied pressures 9.0, 10.8, 12.5, 17.1, 23.2, 25.5, and 29.6 GPa. **b** The in-plane electrical resistivity of FePSe_3_ single crystal as a function of temperature, and applied pressures 29.6, 33.6, 38.0, and 41.4 GPa. Insets of **a**, **b** show the enlarged low-temperature parts of the measured resistivity. Line colors are the same as those used in **a**, **b**. Inset of **b** shows the photograph of a single-crystal FePSe_3_ inside a DAC for resistivity measurements. The scale bar is 100 μm. **c** The temperature dependence of the electrical resistivity of FePSe_3_ at 29.6 GPa under magnetic fields of 0, 0.1, 0.25, 0.5, 1, 2, and 5 T. Inset shows the field dependence of *T*_c_ for FePSe_3_ at 29.6 GPa. **d** Temperature–pressure phase diagram of FePSe_3_, where solid circles represent the pressure dependence of the onset superconducting transition temperatures (5*T_c_), and the blue triangles represent the pressure dependence of Hall coefficient. *T*_N_ represents the Neel temperature of FePSe_3_

Figure 4a presents the resistance *R* of FePSe_3_ as a function of temperature *T* for pressure in the range of 9.0–29.6 GPa. A sharp drop of *R* at an onset temperature of ~2.5 K is clearly observed near the critical pressure *P*_c_ ≈ 9.0 GPa, which is just above the pressure-driven structural phase transition point and indicates the occurrence of SC within the HP phase of FePSe_3_. During further pressure increase, the onset *T*_c_ suddenly increases to 5.5 K above 20 GPa, while the transition becomes much broader. The superconducting transition becomes sharper with zero resistivity reached at 3 K around 30 GPa. Above 29.6 GPa, the *T*_c_ of FePSe_3_ shows a slight tendency to decrease with the pressure increase, but the SC state remains up to 41.4 GPa (the highest pressure in our measurements, as shown in Fig. 4b). The zero resistance has been reached in the experiment for most of the pressure points. All these evidences highlight the fact that the emerging SC state is an intrinsic behavior of the HP phase of FePSe_3_.

We have also applied magnetic field to suppress the SC. Under the constant pressure of 29.6 GPa, temperature dependences of resistance of FePSe_3_ at various magnetic fields are shown in Fig. 4c. During the increase of the magnetic field, the transition is gradually suppressed, which further confirms that it is the superconducting transition. We have obtained the upper critical field (*H*_c2_) as shown in the inset of Fig. 4c. The *H*_c2_ shows an upturn curve, which may be related to the multiband SC in a 2D system. The *T*_c_ values obtained from the above measurements along with the structural and spin-state transitions are plotted in the temperature–pressure phase diagram of FePSe_3_, as shown in Fig. 4d. The application of HP drives the phase transition from the LP, HS, and magnetic insulator (or semiconductor) state to a HP, LS, nonmagnetic, and metallic state in FePSe_3_. SC emerges with a starting *T*_c_ ~ 2.5 K at 9.0 GPa and a maximum *T*_c_ ~ 5.5 K at 29.6 GPa and sustains within the HP phase of FePSe_3_ up to 40 GPa. The SC originated from the parent AFM materials has strong similarities with the unconventional superconductors such as high-*T*_c_ cuprate SCs, FeSCs, and heavy Fermion SCs. These findings demonstrate a successful exploration of new nonmagnetic phases with SC via pressure-driven spin-quenching. The positive Hall coefficient measured at 10 K indicates that holes are the main carriers in the SC phase. The sudden increase of *T*_c_ above 20 GPa, coincident with the anomaly of the Hall coefficient, indicates a possible Fermi surface reconstruction.

## Discussion

**Fig. 5:**
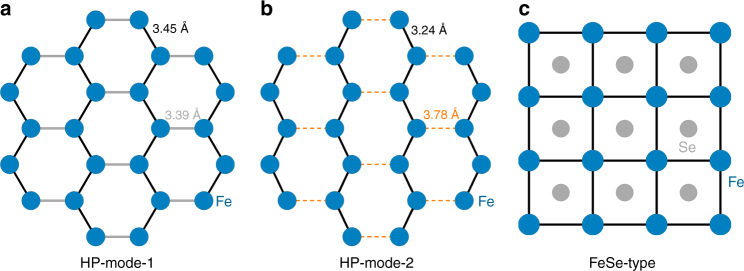
High-pressure structure modes of Fe^2+^ honeycomb lattice and comparison with FeSe-type tetragonal Fe^2+^ lattice. **a** The proposed HP-mode-1 for FePSe_3_ with slightly distorted Fe^2+^ honeycomb lattice. **b** The proposed HP-mode-2 for FePSe_3_ with seriously distorted Fe^2+^ honeycomb lattice. The shortest or the near-shortest Fe–Fe bond lengths are highlighted by black and gray lines in the HP-mode-1 and black and yellow lines in the HP-mode-2, respectively. **c** Tetragonal Fe^2+^ lattice in most Fe-based high-*T*_c_ superconductors with FeSe-type layered structures

The honeycomb layers of FeP*X*_3_ can survive through the pressure-driven quasi-isostructural phase transition. However, the in-plane lattice collapse is proved to be anisotropic and the symmetry breaking down to pseudo-trigonal monoclinic is expected to happen, as shown in Fig. 5a, b. First, the formation of shorter Fe–Fe intermetallic bonding favors the occurrence of cooperative SCO and is consistent with the IMT scenario^27^. Second, the results of in situ-extended X-ray absorption fine structure measurements on Fe K-edge support the HP-mode-2, where the Fe–Fe bonds split into two groups: an intermetallic bond with the Fe–Fe distance of 3.24 Å and an elongated Fe–Fe distance of 3.78 Å, as shown in Supplementary Fig. 5. Therefore, the HP phases of FeP*X*_3_ adopt exactly a distorted honeycomb-like Fe^2+^ lattice. The valence of iron is verified to be +2 in the HP phase of FePSe_3_ (Supplementary Fig. 6), and the increases of the pre-edge peaks indicate the distortion of Fe*X*_6_ octahedra along with the formation of Fe–Fe intermetallic bonding. Figure 5c presents the typical tetragonal lattice of FeSe, which is adopted by all the high-*T*_c_ FeSCs discovered so far. The HP phase of FePSe_3_ with an Fe^2+^ honeycomb lattice presents a unique example of FeSCs without a FeSe-type tetragonal or tetragonal-like lattice. Obviously, the discovery of 3*d* TM-based SC in a new structure prototype will greatly arouse the passion of pursuing high-*T*_c_ SCs.

We have made significant efforts to determine the HP crystal structures of FeP*X*_3_, including repeating powder and single-crystal XRD measurements. However, the detailed structural features, such as the exact space group (symmetry) and atomic positions, are still not well-determined currently due to the difficulty of handling the less than perfect-quality data obtained under HP conditions. The HP crystal structure requires more efforts to be precisely determined in future investigations. In our experiments, SC is only observed in FePSe_3_. The HP phase of FePS_3_ may be either non-superconducting or with a very low *T*_c_ beyond our measurement capability (below 2 K). Moreover, the determination of a detailed *P*–*T* phase diagram of FeP*X*_3_ involving the AFM transition temperatures is now in progress, which may provide more evidence to the SC mechanism within an Fe^2+^ honeycomb lattice and most importantly whether it is an unconventional SC or not. We would also point out that the SC, the route to SC, and the unique Fe^2+^ lattice in FePSe_3_ are obviously different from those in superconducting, nonmagnetic iron metal under compression^30^. There are many other candidates with low-dimensional Fe^2+^ lattices or 3*d* TM-based low-dimensional lattices with *d*^6^ electronic configuration. In principle, they can be tuned to superconducting phases via pressure-driven SCO. Modern structure design and theoretical prediction could facilitate the exploration of such new high-*T*_c_ SCs based on this principle.

## Methods

### Material syntheses

All chemicals were of reagent-grade quality. They were purchased from commercial sources and used as received. FePS_3_ and FePSe_3_ powders were synthesized by heating stoichiometric Fe, P, and S/Se powders at 700 °C in sealed quartz tubes for 24 h. High-quality single crystals were grown by chemical vapor transport method in a two-zone furnace^31^. For both compounds, the temperatures of the two zones were 720–700 °C with iodine as the transport medium. The growth experiments were carried out for a week, and the resulting single crystals were platelets of ~0.01–0.1 mm in thickness and ~0.5–1 mm^2^ in size with black appearance.

### High-pressure structural characterizations

A symmetric DAC with a pair of 300 μm diameter culet-sized diamond anvils was used for all the in situ measurements under HP. Typically, a steel gasket was pre-indented to 40 μm in thickness and a 120 μm diameter hole was laser-drilled to serve as the sample chamber. FePS_3_ and FePSe_3_ powders were pre-compressed into pellets before use. In all the experiments, silicone oil was used as the pressure-transmitting medium (PTM). Ruby balls were used as the pressure gauge and the pressures were calibrated according to the ruby fluorescence method^32^. The in situ XRD measurements were performed at 16 BM-D station of the High Pressure Collaborative Access Team (HPCAT) at Advanced Photon Source (APS) of Argonne National Laboratory (ANL). A focused monochromatic X-ray beam with ~5 μm in diameter (full width at half maximum) and 0.3100 Å wavelength was used, and the diffraction patterns were recorded by using a MAR345 image plate.

### High-pressure spectral studies

HP XES experiments were performed at the 16 ID-D station of HPCAT. Be gaskets were used to confine and pressurize the sample and silicone oil was used as the PTM. X-ray absorption spectrum (XAS) measurements at the Fe K-edge were performed at the 20 ID-C, APS, ANL. A pair of nano-diamond anvils was used to avoid the diffraction glitches from regular single-crystal diamonds. No PTM was used to ensure the uniformity of the thickness.

### High-pressure transport measurements

Electrical resistances of FePS_3_ and FePSe_3_ powders were measured using a four-point-probe resistance measurement system consisting of a Keithley 6221 current source, a 2182A nanovoltmeter, and a 7001 voltage/current switch. Cubic boron nitride layers were inserted into the DAC between the steel gasket and diamond anvils to provide electrical insulation. Four gold wires were arranged to contact the surface of the sample in the chamber.

We conducted the HP electric transport measurements on a FePSe_3_ single crystal under pressure by using the miniature diamond anvil cell^33^. Diamond anvils with 300 μm culet and *c*-BN gasket with sample chambers of diameter 140 μm were used. A FePSe_3_ single crystal was cut with the dimensions of 60 × 60 × 10 μm^3^. Four Pt wires were adhered to the sample using the silver epoxy. Daphne oil 7373 was used as the PTM. Resistance and Hall coefficient were measured using the Quantum Design PPMS-9.

### Data analyses

The 2D XRD patterns were integrated using the Dioptas program^34^. Unit cell parameter refinements were performed with FULLPROF program^35^. The quantitative analyses of the XES data were carried out through IAD (IAD method)^29^.

### Data availability

All data supporting the findings of this work are available from the corresponding author on request.

## Electronic supplementary material

Supplementary Information
