# Supplementary material for: Emergent superconductivity in an iron-based honeycomb lattice initiated by pressure-driven spin-crossover
Source: Nat Commun. 2018 May 15;9:1914. doi: 10.1038/s41467-018-04326-1 (PMC5953925; doi:10.1038/s41467-018-04326-1)
Supplement: Supplementary file 1 — Supplementary Information [file 41467_2018_4326_MOESM1_ESM.pdf]

Supplementary Information for:

Emergent Superconductivity in an Iron-Based Honeycomb Lattice Initiated by Pressure-Driven Spin-Crossover

Wang et al.

Supplementary Figures:

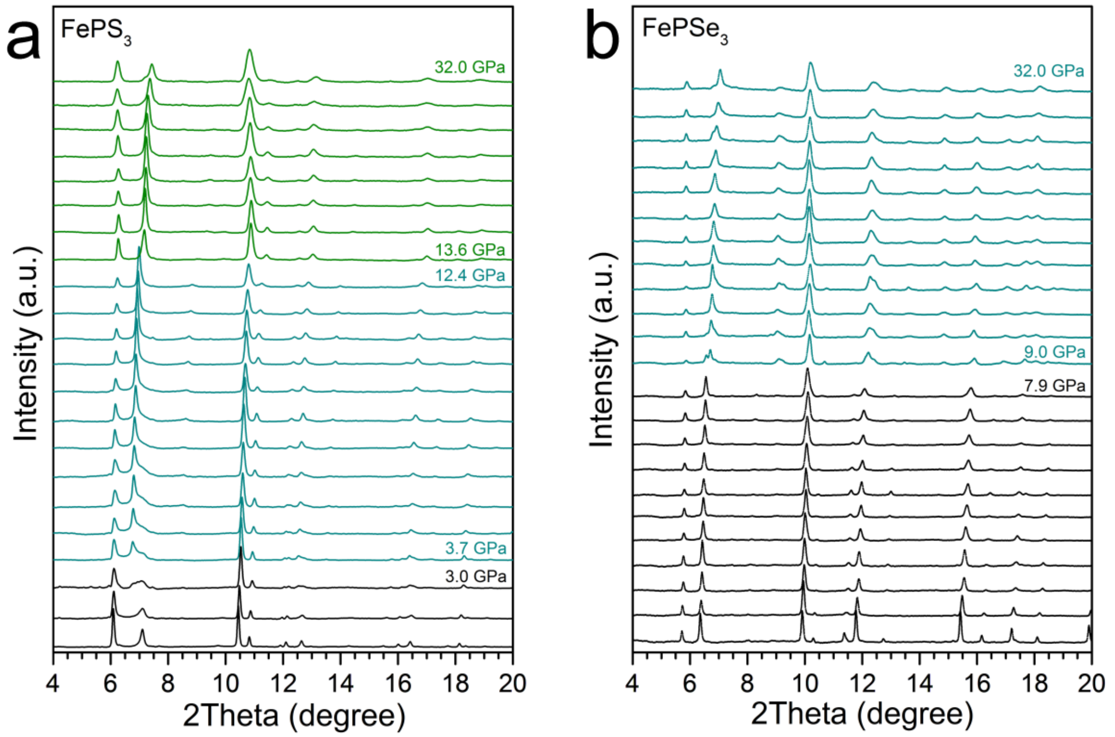

Supplementary Figure 1. In situ XRD patterns of FePX_3 under compression. **a** XRD patterns of $FePS_3$ under high pressure up to 32.0 GPa. **b** XRD patterns of $FePSe_3$ under high pressure up to 32.0 GPa

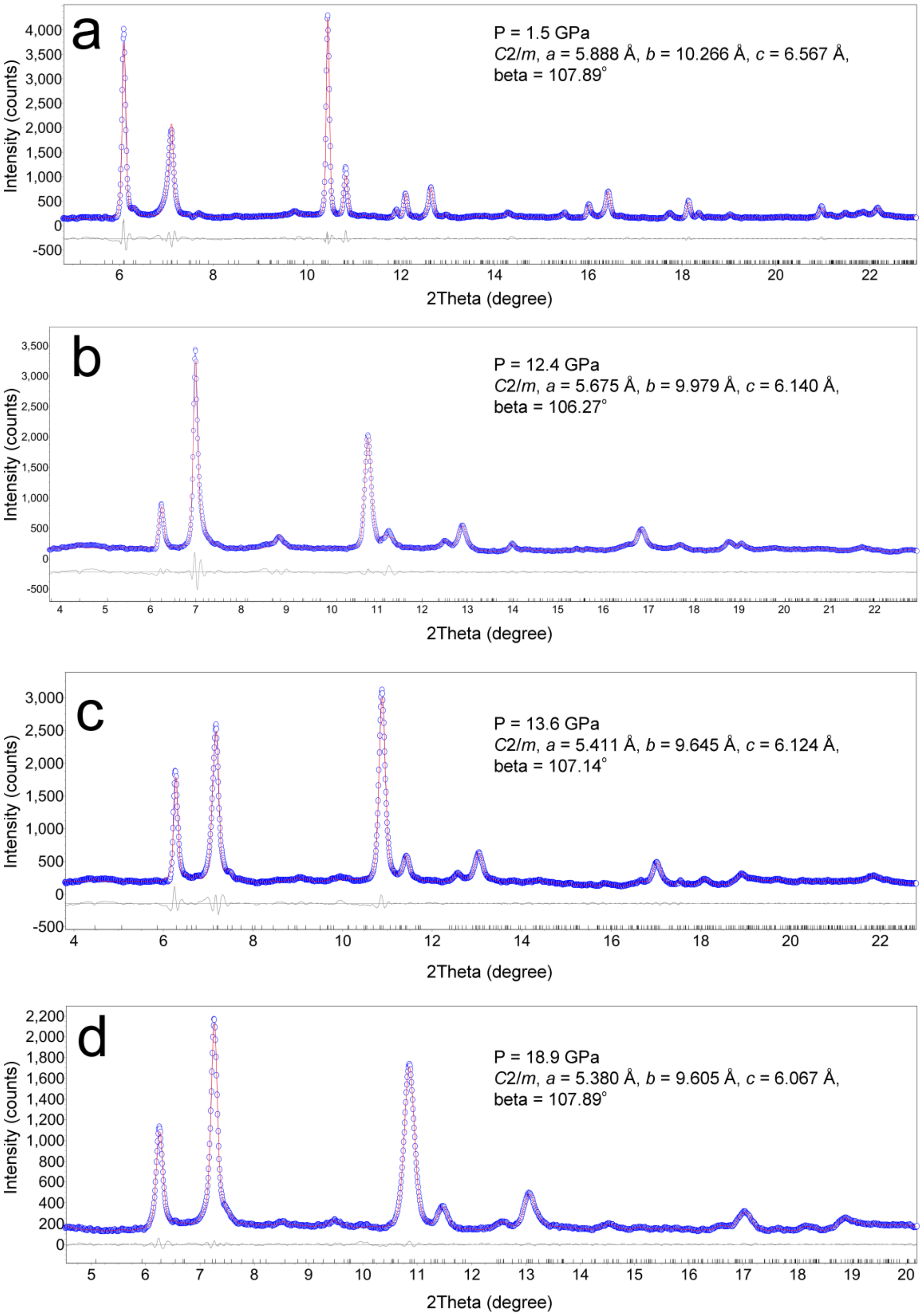

Supplementary Figure 2. Le Bail fitting results for $FePS_3$ under compression. **a-d** The XRD refinement results for $FePS_3$ at pressures of 1.5, 12.4, 13.6 and 18.9 GPa, respectively

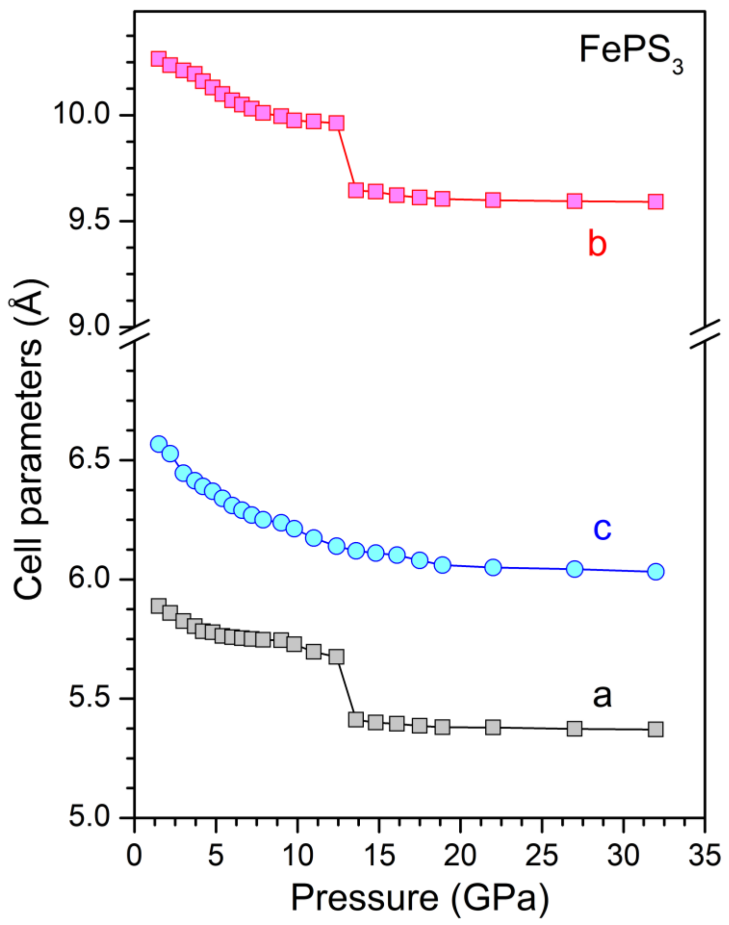

Supplementary Figure 3. The cell parameters of $FePS_3$ under compression

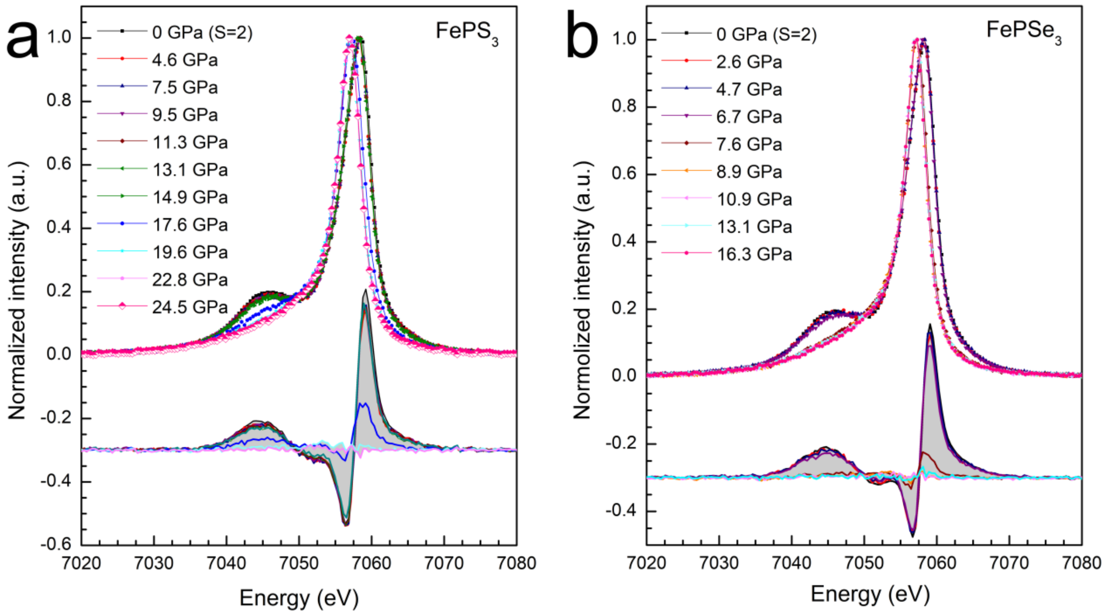

Supplementary Figure 4. XES analyses on the spin state of Fe in $FePX_3$ under high pressure. **a** The raw data and the differential intensity profiles of $FePS_3$ at pressures up to 24.5 GPa. **b** The raw data and the differential intensity profiles of $FePSe_3$ at pressures up to 16.3 GPa. The integrals of the absolute values of the difference spectra (IAD) show the abrupt high-spin (*S*=2) to low-spin (*S*=0) transitions for $FePS_3$ and $FePSe_3$ at 13 GPa and 7 GPa, respectively

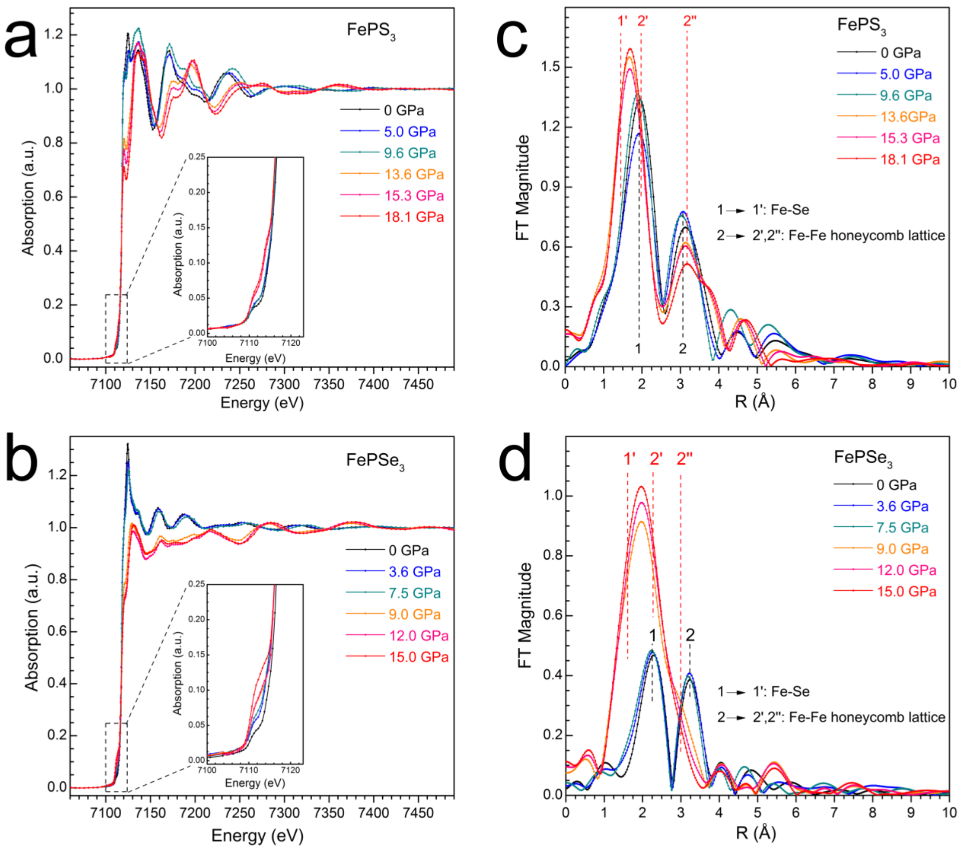

Supplementary Figure 5. EXAFS analyses on the local structure of FePX_3 under compression. **a, b** The raw X-ray absorption spectra (XAS) on the Fe K-edge for $FePS_3$ and $FePSe_3$ as a function of pressure. **c**, **d** Fourier transformed profiles for the Fe coordination environments in $FePS_3$ and $FePSe_3$ as functions of pressure

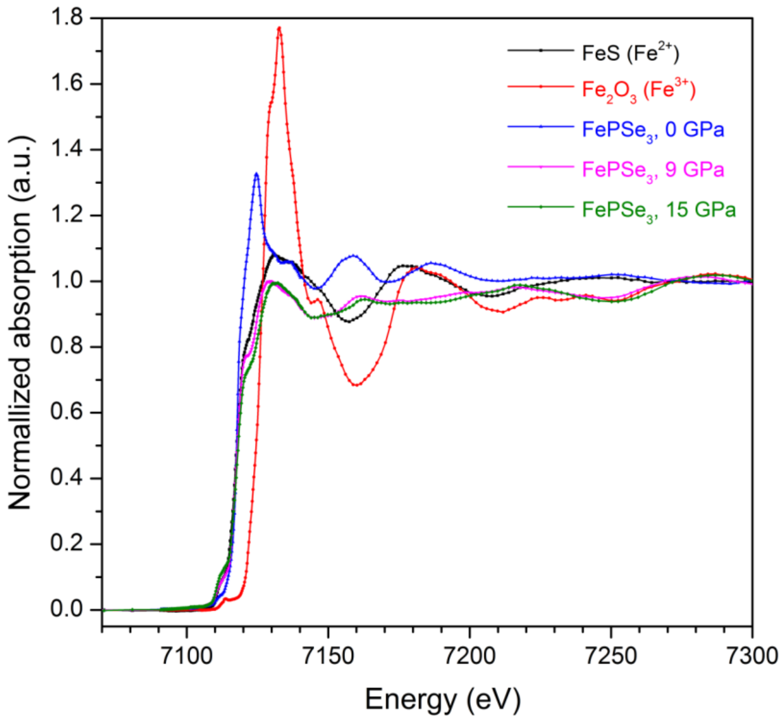

Supplementary Figure 6. XAS data of $FePSe_3$ as a function of pressure. The XAS of FeS and Fe_2O_3 are used as references
